# Real-Time N_2_O Gas Detection System for Agricultural Production Using a 4.6-μm-Band Laser Source Based on a Periodically Poled LiNbO_3_ Ridge Waveguide

**DOI:** 10.3390/s130809999

**Published:** 2013-08-05

**Authors:** Akio Tokura, Masaki Asobe, Koji Enbutsu, Toshihiro Yoshihara, Shin-nosuke Hashida, Hirokazu Takenouchi

**Affiliations:** 1 NTT Photonics Laboratories, Nippon Telegraph and Telephone Corporation, 3-1, Morinosato Wakamiya, Atsugi-shi, Kanagawa Pref. 243-0198, Japan; E-Mails: enbutsu.koji@lab.ntt.co.jp (K.E.); takenouchi.hirokazu@lab.ntt.co.jp (H.T.); 2 Environmental Science Research Laboratory, Central Research Institute of Electric Power Industry, 1646 Abiko, Abiko-shi, Chiba Pref. 270-1194, Japan; E-Mails: yoshiha@criepi.denken.or.jp (T.Y.); shashida@criepi.denken.or.jp (S.H.)

**Keywords:** periodically poled LiNbO_3_ (PPLN), laser source, mid infrared, nitrous oxide (N_2_O), WMS, gas monitoring, cultivated plant, fertilization

## Abstract

This article describes a gas monitoring system for detecting nitrous oxide (N_2_O) gas using a compact mid-infrared laser source based on difference-frequency generation in a quasi-phase-matched LiNbO_3_ waveguide. We obtained a stable output power of 0.62 mW from a 4.6-μm-band continuous-wave laser source operating at room temperature. This laser source enabled us to detect atmospheric N_2_O gas at a concentration as low as 35 parts per billion. Using this laser source, we constructed a new real-time *in-situ* monitoring system for detecting N_2_O gas emitted from potted plants. A few weeks of monitoring with the developed detection system revealed a strong relationship between nitrogen fertilization and N_2_O emission. This system is promising for the *in-situ* long-term monitoring of N_2_O in agricultural production, and it is also applicable to the detection of other greenhouse gases.

## 1. Introduction

Nitrous oxide (N_2_O) is one of the main greenhouse gases (GHGs). N_2_O exhibits a strong greenhouse effect, even though its concentration in the atmosphere (325 parts per billion in volume (ppbv) as measured in 2012) is low compared with carbon dioxide (397 parts per million in volume (ppmv)) or methane (1,830 ppbv) [1]. The atmospheric N_2_O concentration has increased almost linearly at a rate of 0.8 ppbv/year for decades. Because N_2_O has a long lifetime in the atmosphere, it will take a long time for its atmospheric concentration to decrease. Furthermore, N_2_O emissions in 2004 were 62.5% higher than those in 1970 and accounted for 7.9% of the total anthropogenic GHG emissions in terms of carbon dioxide equivalents [2]. It is reported that agriculture and animal husbandry are major contributors as regards anthropogenic N_2_O emissions [2,3]. Agriculture accounted for approximately 60% of the total global anthropogenic emission of N_2_O in 2005, largely through emissions from agricultural soil in which nitrogen fertilizer had been used. Projections indicate that there will be a significant increase in the amount of N_2_O emitted from agricultural soil through to 2030 as a result of the expansion in food production [2,3]. At least some of the N compounds in soil, such as nitrate (NO_3_**^−^**) and ammonium (NH_4_^+^), are processed through the bacterial respiratory reactions of nitrification and/or denitrification, by which NO_3_**^−^** and NH_4_^+^ are converted to N_2_O [4]. Finding a way to improve the efficiency of nitrogen use in agricultural production is a key to the mitigation of N_2_O emissions. Direct measurements of the N_2_O emissions from food-production sectors are urgently required.

Gas chromatography has been widely used to measure N_2_O emissions during agricultural production. Although gas chromatography is a well-established method, gas-chromatographic measurement involves many time-consuming procedures. The consumption of carrier gas and make-up gas needed to measure many samples using this method results in high running costs. Moreover, gas chromatography is subject to systematic errors caused by gas sampling. It is difficult to realize automatic sampling and measurement with this method. For the highly sensitive long-term monitoring of cultivated plants *in-situ*, it is desirable for the monitoring system to measure gas concentrations automatically at a constant frequency. Laser absorption spectroscopy is compatible with an automatic measurement system. To develop an automatic monitoring system for laser absorption spectroscopy, gas sampling should be optimized for plants. Collecting gas emitted from plants is difficult because it involves closing the sampling pot to accumulate the emitted gas in the pot, followed by gas extraction from the closed pot. The gas contains a huge amount of moisture due to plant transpiration, and the electronics and optics used in the system are susceptible to moisture damage. In addition, pressure variations in a sampling pot and a lack of air ventilation caused by gas sampling disturb the cultivation environment.

N_2_O exhibits its strongest absorption band in the 4.6-μm region [5]. Therefore, a 4.6-μm-band CW laser source is suitable for continuous emission monitoring. For these applications, mid-infrared (mid-IR) laser sources based on difference-frequency generation (DFG) in quasi-phase-matched (QPM) lithium niobate (LiNbO_3_ (LN)) are promising because they can provide a continuous-wave (CW) mid-IR laser in the 2 to 5 μm wavelength range and operate at room temperature. A high conversion efficiency can be achieved by using a waveguide structure, so we can obtain sufficient mid-IR output power [6–10]. While monitoring the concentration of atmospheric N_2_O requires a sensitivity as low as about sub ppbv, a detection limit as low as a few tens of ppbv is applicable for N_2_O monitoring during agricultural production. A laser source based on QPM-LN has advantages other than sensitivity, which are derived from the stable material and the use of reliable near-IR lasers.

In this work, we obtained a QPM-LN waveguide laser source with a high conversion efficiency for N_2_O gas detection. Using the laser source, we have developed a N_2_O monitoring system that automatically measures the concentrations of gases emitted by potted plants. We also report the variations in the concentrations of N_2_O gases emitted by a cultivated tomato plant.

## 2. Mid-IR Light Generation using QPM-LN Waveguide

Light in the 4.6-μm band is generated by wavelength conversion based on DFG, which is a second-order nonlinear optical effect. For the DFG, a difference-frequency light, called an idler light, is generated from two input lights, which are called pump and signal lights. When a pump light with a wavelength λ_p_ = c/ν_p_ and a signal light with a wavelength λ_s_ = c/ν_s_ are launched into a nonlinear optical crystal, an idler light with a wavelength λ_i_ = c/ν_i_ (ν_i_ = ν_p_ − ν_s_) is generated by the DFG process, where c is the velocity of light and ν is its frequency. Thus, this can be expressed using wavelengths as:

(1) $$\frac{1}{\lambda_{i}}=\frac{1}{\lambda_{\text{p}}}-\frac{1}{\lambda_{\text{s}}}$$

As shown in Equation (1), various mid-IR light wavelength ranges can be generated simply by choosing commercially available near-IR laser diodes (LDs) as appropriate pump and signal lights. Near-IR telecommunication LDs have stable operation characteristics and exhibit reliable performance for a long period. Mid-IR CW output at room temperature is readily available since near-IR pump and signal LDs with CW operation can be easily obtained. A stable wavelength sweep can be obtained for optical absorption measurement because mode hopping is less likely to happen during the wavelength sweep. Additionally, the pump or signal outputs from near-IR LDs can be used effectively for an optical alignment instead of a mid-IR output, which is difficult to visualize. Efficient wavelength conversion is achieved by satisfying the phase-matching condition (Δβ = 0) using a quasi-phase-matching technique, where the phase mismatch Δβ is defined by:

(2) $$\Delta \beta =2\pi \left(\frac{{\text{n}}_{\text{p}}}{\lambda_{\text{p}}}-\frac{{\text{n}}_{\text{s}}}{\lambda_{\text{s}}}-\frac{{\text{n}}_{\text{i}}}{\lambda_{\text{i}}}-\frac{1}{\Lambda }\right)$$

In this equation, Λ is the periodic poling period, and n_p_, n_s_, and n_i_ are the respective refractive indices at wavelengths of λ_p_, λ_s_, and λ_i_. Because Λ is a design parameter, modification to various wavelength combinations is easily accomplished. On the basis of a small signal approximation, where the attenuation of the two input light powers P_p_ and P_s_ is negligible, the converted light power P_i_ is given by:

(3) $${\text{P}}_{\text{i}}=\frac{\eta {\text{P}}_{\text{p}}{\text{P}}_{\text{s}}}{100}$$

where η (%/W) is the conversion efficiency and is given by:

(4) $$\eta =\eta_{\max}{\text{sinc}}^{2}\left(\frac{\Delta \beta \text{L}}{2}\right)$$

and:

(5) $$\eta_{\max}\propto \frac{{\text{L}}^{2}}{{\text{A}}_{\text{eff}}}$$

**Figure 1.**
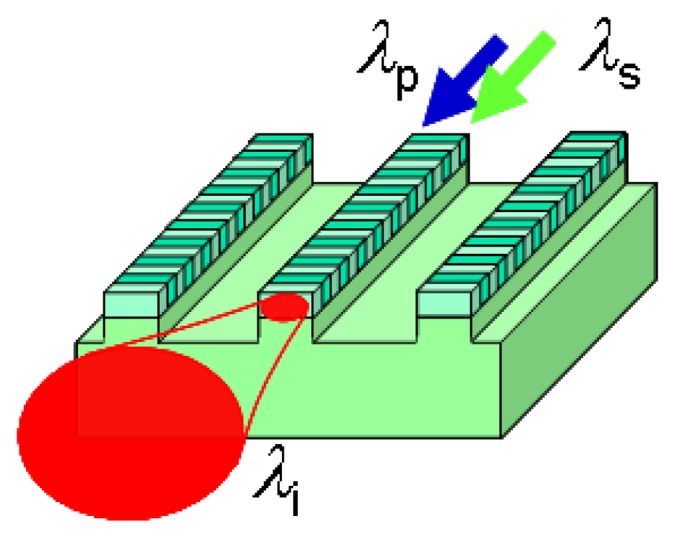
Structure of QPM-LN waveguide device.

The conversion efficiency η becomes η_max_ when the phase mismatch is zero. The power of the converted light P_i_ increases in proportion to input powers P_p_ and P_s_. Furthermore, P_i_ is proportional to the square of device length L and the inverse of the effective interaction cross-section A_eff_. To reduce A_eff_ and thereby achieve efficient wavelength conversion, it is effective to use a waveguide structure that confines the three interacting lights in a small core area. The waveguide structure also assists the extension of the device length L, because the lights can propagate in the waveguide over a long distance without spreading out. A DFG device using a QPM-LN waveguide is shown schematically in Figure 1.

## 3. Experimental Section

### 3.1. QPM-LN Waveguide Fabrication

**Figure 2.**
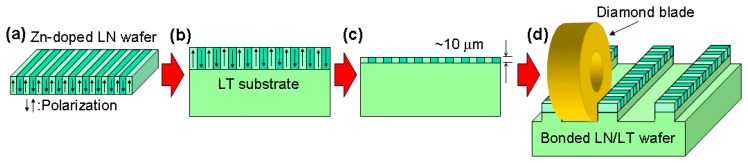
Process for fabricating direct-bonded ridge waveguide: (**a**) electrical poling; (**b**) direct bonding; (**c**) lapping and polishing; (**d**) waveguide fabrication using dicing saw.

Our QPM-LN waveguide fabrication method is based on direct bonding [11]. The fabrication process is shown in Figure 2. We used Zn-doped LN as a core layer and lithium tantalate (LiTaO_3_ (LT)) as a cladding layer. First, we made a periodically poled structure on an LN wafer by using a conventional electrical poling method. We then brought two wafers into contact in a clean atmosphere and annealed them at around 500 °C to achieve complete bonding. Next, the thickness of the core layer was reduced to around 10 μm by lapping and polishing. Then, the ridge structure was fabricated by cutting grooves using a dicing saw. The waveguide end faces were cut at an angle using a dicing saw and coated with antireflection film to prevent undesired back reflection. The Zn-doped LN core layer makes our waveguide highly resistant to photorefractive damage. Moreover, because the direct bonding technique does not use any adhesives, the fabricated waveguide is transparent in the mid-IR range and has better long-term reliability than those made using adhesives. This fabrication process is described in detail elsewhere [6,10].

### 3.2. DFG Performance and Experimental Setup for Laser Source Evaluation

The fabricated waveguide was assembled in a fiber pigtail module package. The module is 12 mm thick, 30 mm wide, and 73 mm long. To excite the transverse magnetic mode of the waveguide, we used a polarization-maintaining fiber as an input fiber. The module has a Peltier element and a thermistor to control the waveguide temperature. The phase-matching wavelength can be tuned by the temperature control. The pump and signal lights from the input fiber were coupled by a set of lenses to prevent heat damage to the connection area at a high input power. The heat damage of this module was low compared with that of modules connected using butt joints reported in previous studies, because light absorption tended to increase the temperature of the adhesive agent used at the butt joints [6–10]. This lens coupling enables us to inject high-power 1.06-μm-band pump light, which is amplified by an ytterbium-doped fiber amplifier (YDFA).

**Figure 3.**
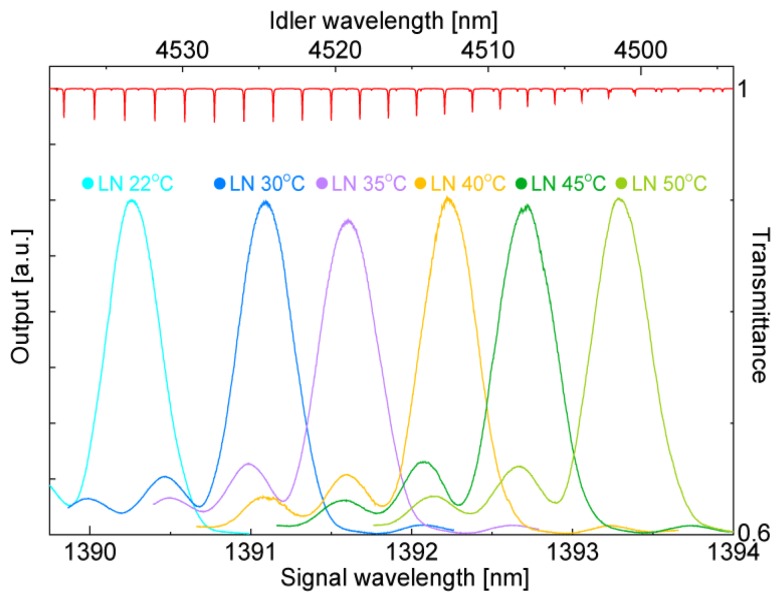
DFG tuning curves at various temperatures.

The DFG tuning curves measured at various LN waveguide temperatures as a function of signal wavelength are shown in Figure 3. The curves were obtained by using a tunable semiconductor laser (Santec Corporation, Aichi, Japan) as a signal light at a pump wavelength of 1.06396 μm. The corresponding N_2_O gas absorption lines calculated from values in the high-resolution transmission molecular absorption (HITRAN) database are also shown at the top of the graph [5]. The wavelength conversion efficiencies were almost constant at a waveguide temperature range of 20 to 50 °C. We could tune the phase-matching wavelength to a suitable absorption line for detection from among more than a dozen lines by controlling the waveguide temperature and the pump and signal wavelengths. Note that the tuning curve does not correspond to the line shape of the mid-IR output, but represents the wavelength range that the waveguide can generate at the waveguide temperature. The line width of the mid-IR idler is estimated from the convolution of the pump line shape and the signal line shape as described in Equation (3). The mid-IR line width in terms of frequency is smaller than the sum of the pump and signal line widths.

**Figure 4.**
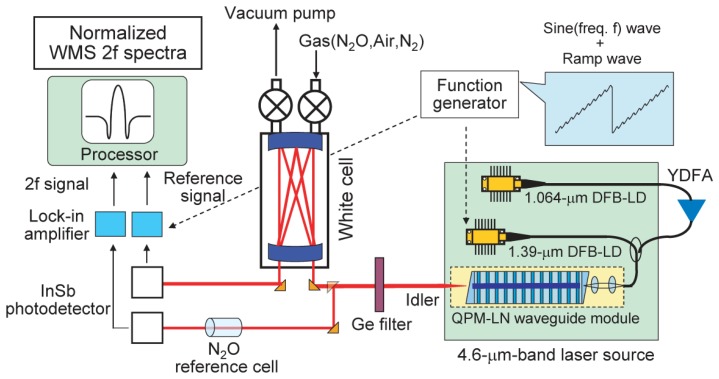
Experimental setup for N_2_O gas detection.

The experimental setup for evaluating the laser source is shown schematically in Figure 4. We fabricated a 4.6-μm-band laser source for detecting N_2_O gas. The source is compact and reliable. It consists of two LDs, a YDFA, a wavelength division multiplexing fiber coupler, and a QPM-LN module. A 1.06-μm-band distributed feedback laser diode (DFB-LD) (Eagleyard Photonics GmbH, Berlin, Germany) with a line width of 2 MHz and a maximum output of 45 mW, and a 1.39-μm-band DFB-LD (NTT Electronics Corporation, Kanagawa, Japan) with a line width of 2 MHz and a maximum output of 45 mW were used to generate the pump light and signal light, respectively. The 1.06-μm-band pump LD covered the 1.3 nm wavelength range associated with operation temperature variation at a given injection current and the 0.2 nm wavelength range associated with an injection current variation of 90 mA at a given operation temperature. The 1.39-μm-band signal LD covered the 2.4 nm wavelength range associated with operation temperature variation at a given injection current and the 0.9 nm wavelength range associated with an injection current variation of 90 mA at a given operation temperature. The mid-IR tuning range in the 4.6-μm band was about 60 nm (30 cm^−1^). For example, when the pump LD and the signal LD cover wavelength ranges of 1,062.6 to 1,064.1 nm and 1,390.0 to 1,393.0 nm, respectively, the mid-IR idler covers a wavelength range of 4,480.03 (2,232.1 cm^−1^) to 4,538.51 nm (2,203.4 cm^−1^). The line width of the mid-IR light was estimated to be <4 MHz (0.00013 cm^−1^) from the pump and signal line widths. The absorption line widths of N_2_O at a pressure of 13.3 kPa in the 4.6-μm band are estimated to be about 600 MHz (0.02 cm^−1^) [5]. The line width of the mid-IR was adequately narrow for the detection of each N_2_O line. We used wavelength modulation spectroscopy (WMS) to achieve highly sensitive detection [12]. By using a function generator, an injection current from a 1.39-μm-band DFB-LD was modulated with a sine wave at around 15 kHz superimposed on a ramp wave at around 1 Hz. By modulating the current of the signal light LD, we were able to obtain stable WMS spectra. We swept the signal wavelength in a small range of 0.2 nm (an injection current of about 20 mA) around the center wavelength as a ramp wave. The main component of the detection equipment was a White cell with a 16- or 10-m optical path. A germanium filter was used to eliminate the unnecessary near-IR pump and signal lights. The mid-IR light output was collimated correctly using optical lenses (not shown in the figure) after the source and was focused before the photodetector. The output light was split by a half mirror before the White cell. The light for the concentration measurement passed through the White cell before it was received by an InSb photodetector. Another light passed through the 0.5%-N_2_O reference cell to normalize the obtained signal. The output signal from the photodetector was detected with a lock-in amplifier. The WMS spectrum consisted of the second-harmonic (2f) component of the modulation frequency, which was derived from the lock-in amplifier. Utilizing the ratio of the White cell signal to the reference cell signal for the conversion from 2f signal to concentration, we exclude the intensity fluctuation of the laser source. The reference cell signal was also used to search for the peak position. As regards the wavelength conversion efficiency, we obtained a CW mid-IR output with a power of 0.62 mW at an internal conversion efficiency of 5.9%/W calculated with a pump power of 507 mW and a signal power of 20.5 mW on the waveguide output side.

## 4. Results and Discussion

### 4.1. N_2_O Spectra and Detection Limit

We evaluated the laser source under the conditions described above. The sample gases for the evaluation were N_2_-diluted N_2_O gas and N_2_-diluted air. We used a White cell with a 16-m optical path. We selected the absorption line at 2,201.75 cm^−1^ (^14^N_2_^16^O P24) to evaluate the laser source to avoid interference from the absorption lines of other gases contained in air, such as water (H_2_O), carbon monoxide (CO), and carbon dioxide (CO_2_). To obtain accurate gas concentration values, it is preferable to perform the N_2_O measurement with the pressure reduced to 13.3 kPa because overlapping adjacent absorption lines are separated without any reduction in the absorption amount.

**Figure 5.**
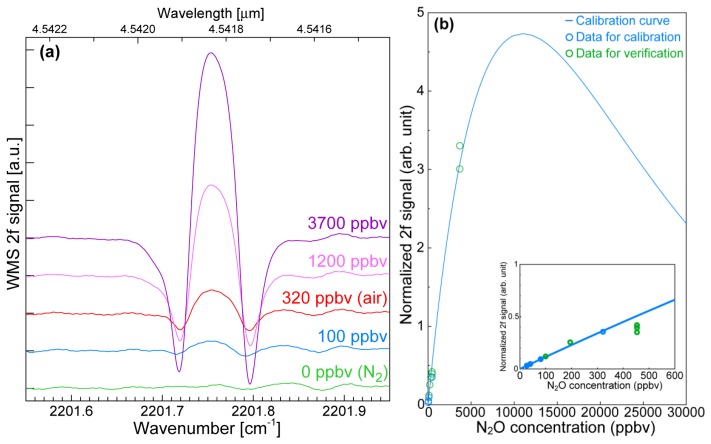
(**a**) WMS 2f spectra at various N_2_O concentrations; (**b**) Calibration curve of N_2_O concentration. The inset shows the same curve in the low concentration range.

The raw data of the N_2_O WMS 2f spectra at various N_2_O concentrations are shown in Figure 5a. The input pump and signal powers were 200 and 40 mW, respectively. The pump and signal wavelengths were 1,065.17 and 1,391.51 nm at injection currents of 170 and 175 mA, respectively. The sweep range of the signal wavelength was 0.2 nm, which corresponded to an injection current of 25 mA. The modulation index for the WMS was 2.8 × 10^−6^, which was 3.3 times larger than the FWHM of the absorption line. The large modulation amplitude contributed to the large 2f signal and thus the high signal to noise (S/N) ratio although the line widths of the obtained spectra were broadened by the large modulation amplitude. We gave priority to the S/N ratio because our conversion did not require a precise absorption line shape as described below. The red and blue lines are spectra obtained at N_2_O concentrations of 320 and 100 ppbv, respectively. These spectra indicate that the atmospheric N_2_O concentration can be clearly detected and that N_2_O concentrations lower than 100 ppbv can be detected with this 4.6-μm-band laser source.

To convert the WMS 2f signal into the N_2_O concentration, we used the WMS 2f peak value from the zero line. Figure 5b shows a calibration curve based on a semi-empirical method and calculated using the parameters in the HITRAN database and a parameter of our system. The fitting function is the second derivative component of the optical transmittance as:

(6) $$\frac{{\text{V}}_{2\text{f White cell}}}{{\text{V}}_{2\text{f White cell}}}=A\alpha \text{c}\cdot \exp\left(-\alpha \text{c}\right)$$

In this equation, c and V_2f_ are the gas concentration and the 2f signal voltage without an offset component, respectively. A is a fitting parameter, and α is a parameter determined by path length and values in HITRAN database. The blue circles are the fitting data and the green circles are the data for verification. The input powers of the plotted data are normalized. The inset shows the curve in the low-concentration range. We defined the detection limit as the maximum noise level in the measured spectra for verification at an input pump power of 200 mW, which means that the limit is the concentration at which the S/N ratio is 1. From the concentration at the crossing point between this curve and the normalized maximum noise level, we found that the detection limit was 35 ppbv, which corresponds to one-tenth of the atmospheric N_2_O concentration. The blue circles for fitting in Figure 5b were measured at an input pump power of 700 mW. Because the high input power contributes to the improved S/N ratio, the detection of the gas concentrations below 35 ppb is reasonable. The upper detection limit is naturally 11,000 ppbv (11 ppmv), and a one-to-one correspondence is obtained at concentrations below. Further work includes verification of the calibration curve at near the upper limit and verification in detail by comparison with results obtained using gas chromatography. This result suggests that this laser source is suitable for *in-situ* N_2_O measurements with a concentration resolution around 30 ppbv. Some sensors using QCL were reported, including a sensor based on photoacoustic spectroscopy, a sensor based on direct absorption using a multiple pass cell, and a sensor based on the WMS method [13–15]. These sensors all achieved high sensitivity at a range from a few ppbv to sub ppbv level. Although the sensitivity of the sensor used in this work was low compared with the above sensors using QCL for ambient N_2_O monitoring, a sensitivity level of a few tens of ppbv was adequate for monitoring in practical agricultural production. On the other hand, the reliable performance of the laser source for a long period and the easy alignment of optical path using a near-IR pump or signal light were strong advantages of the sensor based on a QPM-LN waveguide for an agricultural environment.

### 4.2. N_2_O Detection in Practical Agricultural Production

We applied the laser source to a system for monitoring N_2_O during agricultural production. A highly sensitive automatic *in-situ* monitoring system should help us to find a way to reduce N_2_O emissions. In this work, we measured the N_2_O emitted from tomato plants cultivated hydroponically on rock wool as a model. In fact, the use of hydroponics with rock wool is a popular approach for practical fruit and vegetable cultivation, and is used to grow, for example, tomatoes, cucumbers, and eggplants. We used another laser source and selected the absorption line at 2,205.69 cm^−1^ (^14^N_2_^16^O P20) to monitor the N_2_O emitted by plants. We selected another line because of the difference between the obtained wavelength ranges of the two DFB-LDs. This line was also chosen to avoid interference from the absorption lines of other gases. The characteristics and detection limit of this laser source were very similar to those of the laser source for evaluation described above. We used a White cell with a 10-m optical path for this monitoring. The input pump and signal powers were 300 and 40 mW, respectively. The pump and signal wavelengths were 1,064.08 and 1,391.40 nm at injection currents of 170 and 141 mA, respectively. The sweep range of the signal wavelength was 0.2 nm, which corresponded to an injection current of 21 mA. The modulation index for the WMS was 2.1 × 10^−6^, which was 2.4 times larger than the FWHM of the absorption line. The modulation amplitude was determined based on the balance between the 2f signal amplitude and the noise level.

**Figure 6.**
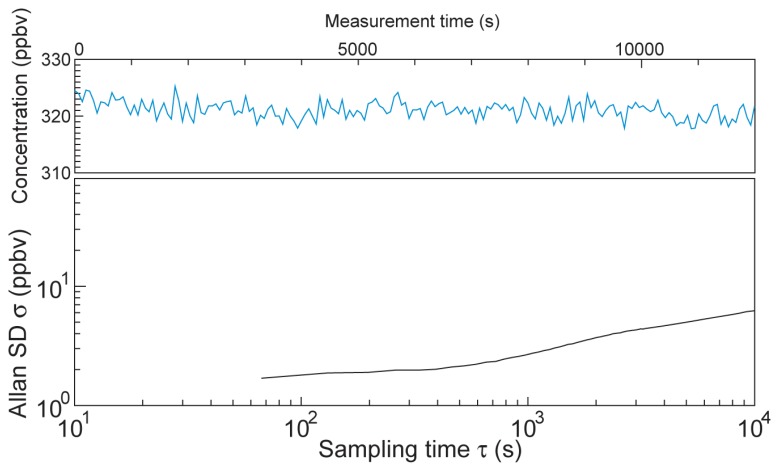
Allan variance plot and concentration variation for N_2_O of air in the laboratory over a 3 h period with a 1 min averaging time.

An Allan variance plot and the concentration variation over a 3 h period with 60-s-averaging data collection are shown in Figure 6 [16]. From the concentration plot in Figure 6, the standard variation σ is 1.5 ppbv, and is below 6.1 ppbv for a longer time (data not shown). Since the measured data were averaged for 60 s, the Allan plot starts from over 60 s. Although the behavior of white noise dominant region is unclear due to lack of data before 60 s, σ_Allan_ at a sampling time range between 70 and 1,000 s is stable at below 3 ppbv. A long sampling period leads to a large σ_Allan_ presumably due to instrumental drift induced by temperature variation. A temperature variation of around a few degrees centigrade influences the optical alignment and detection sensitivity of the photodetector. An evaluation of the stability in a short time range and stability improvement are challenges for future work.

**Figure 7.**
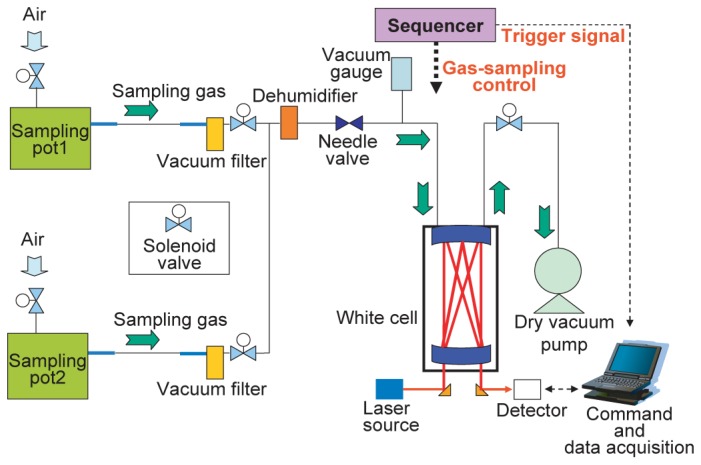
Schematic diagram of proposed monitoring system.

The N_2_O emissions from a pot were captured using a closed-chamber technique [17]. A Wagner pot (1/2000a, 252 mm in diameter × 300 mm in height; Fujiwara Science Co. Ltd., Tokyo, Japan) with another mass of rock wool (20 cm layer) laid at the bottom was used as a pot. The pot had a hinged lid that allowed it to be opened and closed. Water and fertilizer were periodically supplied to the plants in the pot via an injection pump. We developed an *in-situ* intermittent gas monitoring system that measured the N_2_O concentrations of sample gases at a specific time interval. This system was developed to automate gas measurement and the gas sampling procedure performed during gas-chromatography measurement [18]. In this work, long-term-variation was examined with one measurement per chamber closure, and by designing a large N_2_O emission rate and a short accumulation period. Figure 7 shows a schematic diagram of the monitoring system. This system enabled us to measure sample gases alternately from two different pots to compare the growth conditions of the plants. The top cover had an air-supply nozzle and a gas-sampling nozzle. Automatic gas-transfer control was achieved with solenoid valves and a needle valve. Vacuum filters and a dehumidifier (Perma pure dryer, MD-110-12p-4; GL Sci. Co. Ltd., Tokyo, Japan) were inserted in the gas line to prevent the penetration of dust and moisture. This permeable membrane type dehumidifier was similar to one used in gas chromatography. The main purpose of introducing the dehumidifier was to prevent the adsorption of moisture inside the solenoid valves and on the mirrors in the White cell. Humidity of more than 80% had to be reduced to at least the atmospheric level. The sample gas was inducted very slowly into a White cell by utilizing the pressure difference between the atmosphere and the cell until the cell pressure approached a specific value. The inducted gas was evacuated into the atmosphere with a dry vacuum pump. The opening and closing of the solenoid valves for the gas sampling was controlled by a sequencer using timers and a vacuum gauge. The reproducibility of the pressure in the White cell was controlled at <±267 Pa (2 Torr) by using the vacuum gauge. The sequencer started a N_2_O concentration measurement by sending a trigger signal to a computer. The WMS 2f peak intensity was converted to a concentration value and stored in the computer. We used 1-ppmv standard N_2_O gas for system calibration in this measurement. We verified the concentration value using a gas-chromatographic determination. When the gas sampling for the gas-chromatographic measurement was carried out immediately after the sampling for this monitoring system, the concentration measured with the system and that obtained by gas chromatography were 2.18 × 10^3^ and 2.22 × 10^3^ ppbv, respectively. The concentration value obtained with the system agreed well with obtained using gas chromatography.

The measurement sequence and time setting used in this measurement are shown in Table 1. The measurement procedure was as follows: First, air ventilation was carried out by opening both the air-supply nozzle and the gas-sampling nozzle to avoid disrupting the growth environment of the tomato plants after gas evacuation. When the N_2_O emission is small, the gas concentration, which is measured as a background (BG) measurement, can be adopted as a reference for ambient air. Next, N_2_O gas was accumulated in the pots by closing both the air-supply nozzle and the gas-sampling nozzle. Then, to purge the gas line, after the gas in the White cell was evacuated, the sample gas was inducted into the cell with the air-supply nozzle closed followed by pumping. We used an air bag to keep the inside of the pot at ambient pressure. The purge process was repeated twice to remove residual gas. Finally, after the gas induction, the solenoid valves on both sides of the cell were closed, and a measurement was started. Then, the cell was evacuated, followed by the purge process for another pot or by returning to the ventilation process. This measurement was carried out every 30 min with a ventilation period of 14 min, a gas accumulation period of 3 min, and a measurement period of 1 min.

**Figure 8.**
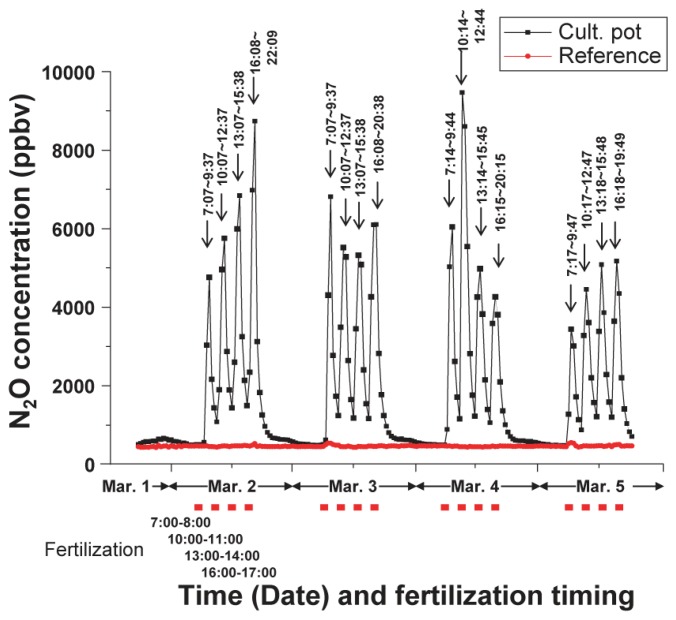
Variation in concentration of N_2_O gases from a tomato plant cultivated hydroponically on rock wool. The black line shows the data from the cultivation pot and the red line shows the data from the reference pot. Black arrows and red symbols indicate peak duration and fertilization timing, respectively.

For at least three consecutive months, this monitoring system could successfully detect concentration variations resulting from the variation in the N_2_O emissions. Figure 8 shows part of the monitoring data, which were obtained over four successive days in early March 2013. In this observation, one pot contained a hydroponically cultivated tomato plant grown under conditions where a fixed quantity of fertilizer was added every 3 h and four times a day, and another pot contained no plant as a reference. The vertical and horizontal axes are the N_2_O concentration, time and the fertilization timing, respectively. The red symbols below the horizontal axis and the black arrows indicate the fertilization timing and peak duration, respectively. The black line shows data from a cultivation pot and the red line shows data from a reference pot that did not contain any plants. The fertilization was conducted four times a day between 7:00 am and 5:00 pm. Approximately 70–80 mL of nutrient solution was dripped for 1 h on each occasion (*i.e.*, total of 280–320 mL of solution was supplied per day). On each occasion, 6.8–7.8 mg of nitrogen was supplied. The fresh weight of the plant during the observation was estimated to be 1.5 to 2 kg from a measurement performed at the end of the experiment. A sharp peak was observed soon after each fertilization while no peak appeared for the period without fertilization. This indicated that the supplied fertilizer was immediately resolved on a time scale of about one hour, and caused a sudden N_2_O emission under this cultivation condition. The N_2_O emission began to increase at least 7 min after the start of each fertilization, and lasted for 3 to 5 h after the end of the daily fertilization. The daily N_2_O emission rates were estimated to range from 8.4% (March 5) to 11.5% (March 4) of the total nitrogen supplied as fertilizer (27.3–31.2 mg-N per day). In addition, N_2_O peak values changed in the 3,500 to 9,500 ppbv range, which is an approximately threefold difference, although the fertilization quantity was the same every time. Such a large deviation in the N_2_O generation would mean changes in the plant physiology related to rhizosphere-microbe activity. For example, solar radiation would affect the water and nutrient uptake by plants. This could consequently affect the N_2_O generation related to microbe activity in the rhizosphere. To investigate the N_2_O emissions that are dependent on the plant growing conditions will constitute the next interesting task. The result indicated that this monitoring system enabled us to detect sudden variations in N_2_O emissions with adequate sensitivity and time resolution for a sufficient period. The result also showed that the monitoring system is very effective for investigating the relationship between growth conditions, especially fertilization, and N_2_O emissions. This simple and stable system contributes to the reduction in the systematic error that occurs during gas sampling. A great merit of the system is that it offers automatic sampling and measurement at a constant duration for a long time.

## 5. Conclusions

We have developed a 4.6-μm-band mid-IR laser source based on DFG for N_2_O gas detection. This is a reliable and compact laser source that uses a QPM-LN waveguide module and two near-IR telecommunications LDs. We obtained a stable CW output at a power of 0.62 mW and an internal conversion efficiency of 5.9%/W as typical properties. We successfully demonstrated N_2_O gas detection with this laser source. We obtained a detection limit of 35 ppbv for N_2_O detection. Using this laser source, we constructed a novel *in-situ* intermittent gas monitoring system for the observation of cultivated tomato plants. The system was optimized for the measurement of gases from living plants. The stable and automatic monitoring system was highly advantageous for the investigation. We detected a sharp peak soon after each fertilization while we could find no peak for periods where there was no fertilization. This monitoring system is very effective for investigating the relationship between cultivation conditions and N_2_O emissions. The DFG-based mid-IR laser source using a QPM-LN waveguide can cover wavelengths up to about 5 μm, to which LN is transparent. This laser source and system are promising for *in-situ* long-term monitoring of N_2_O and other GHGs in agricultural production.

## Figures and Tables

**Table 1.** Measurement sequence and time setting in this monitoring.

**Meas. Type**	**Process**	**Time**
	Pumping	1 min
	Air ventilation	14 min
	Air induction from both pots	1 min
BG	Measurement	1 min
	Gas storage (in the pots)	3 min
	Pumping	1 min
	Purge (induction from pot1)	1 min
	Purge (pumping)	1 min
	Air induction from pot1	1 min
Pot1	Measurement	1 min
	Pumping	1 min
	Purge (induction from pot2)	1 min
	Purge (pumping)	1 min
	Air induction from pot2	1 min
Pot2	Measurement	1 min
